# The role of the age and gender, and the complexity of the syntactic unit in the perception of affective emotions in voice

**DOI:** 10.1590/2317-1782/20242024009en

**Published:** 2024-07-19

**Authors:** Baiba Trinite, Anita Zdanovica, Daiga Kurme, Evija Lavrane, Ilva Magazeina, Anita Jansone

**Affiliations:** 1 Voice and Speech Research Laboratory, Riga Technical University Liepaja Academy – RTU LA - Liepaja, Latvia.

**Keywords:** Voice, Perception, Emotional Prosody, Affective Emotions, Gender, Age, Word, Phrase, Text

## Abstract

**Purpose:**

The study aimed to identify (1) whether the age and gender of listeners and the length of vocal stimuli affect emotion discrimination accuracy in voice; and (2) whether the determined level of expression of perceived affective emotions is age and gender-dependent.

**Methods:**

Thirty-two age-matched listeners listened to 270 semantically neutral voice samples produced in neutral, happy, and angry intonation by ten professional actors. The participants were required to categorize the auditory stimulus based on three options and judge the intensity of emotional expression in the sample using a customized tablet web interface.

**Results:**

The discrimination accuracy of happy and angry emotions decreased with age, while accuracy in discriminating neutral emotions increased with age. Females rated the intensity level of perceived affective emotions higher than males across all linguistic units. These were: for angry emotions in words (z = -3.599, p < .001), phrases (z = -3.218, p = .001), and texts (z = -2.272, p = .023), for happy emotions in words (z = -5.799, p < .001), phrases (z = -4.706, p < .001), and texts (z = -2.699, p = .007).

**Conclusion:**

Accuracy in perceiving vocal expressions of emotions varies according to age and gender. Young adults are better at distinguishing happy and angry emotions than middle-aged adults, while middle-aged adults tend to categorize perceived affective emotions as neutral. Gender also plays a role, with females rating expressions of affective emotions in voices higher than males. Additionally, the length of voice stimuli impacts emotion discrimination accuracy.

## INTRODUCTION

The primary function of the voice is to convey a message that includes linguistic and paralinguistic components. Voicing is a dominant carrier of the message in speech, singing, and animal vocalization^(1)^. Voice plays a crucial role in communication as it transmits nonverbal cues about the speaker's emotions, physical condition, social standing, age, and gender. Prosodic features are one of the main tools for delivering the explicit content of the message, as well as the specific affective dimension that affects the listener’s impression-forming process^(2)^. Scherer et al.^(3)^ suggest that expressing emotions through the voice may be one of the most complex systems of communication.

Communication quality depends on how the speaker's message, including linguistic and paralinguistic layers, is perceived. One could maintain that decoding, or the perception of the voice, is the final output of the encoding process of voice production. Auditory perception and sound carrier modulations are essential in voice signal transmission^(1)^. Through auditory perception, we are able to perceive the emotional expression coded in the voice^(3)^. However, the correct recognition of vocal emotion relies on sharing the same knowledge about what a vocal emotion sounds like.

From a research perspective, emotion is a complex construct that can be defined as consisting of several distinctive components: subjective experience, neurophysiological response patterns mediated via the central and autonomic nervous system, and motor expressions through the voice, face, and gestures, as well as appraisal of the situation and action preparation^(4)^. Physiological arousal, triggered by an emotional stimulus, can manifest as affective prosody – the vocal expression of emotions in speech through intentional and unintentional alterations of the acoustic properties of the speaker's voice^(5)^.

### Encoding of affective prosody

The complete cycle of communicating vocal emotions consists of several distinctive processes: encoding, transmission, and decoding^(6)^. The voice consists of a perceptible external physical signal that expresses the internal emotional process^(3)^. One of the most common approaches in measuring the encoding of affective prosody takes advantage of several acoustical properties (e.g., amplitude and frequency) in order to elucidate the perceptual dimensions of loudness, pitch, speech rate, timbre, and voice quality^(4)^. An increased mean intensity and fundamental frequency (F_0_) and differences in the F_0_ range and F_0_ variability have been observed during both angry and happy speech^(4)^. In general, happy vocalizations tend to have considerable variability in loudness and pitch, in addition to a high pitch and high first two formant frequencies^(7)^, while negative emotions have been most commonly associated with high speech intensity^(6)^.

### Decoding of affective prosody

Contrary to objective measurements of encoded stimuli, vocal affect decoding studies use predominantly subjective methods. Typically, naïve listeners are requested to recognize the emotional features of a given voice sample and indicate the most appropriate emotional label for each stimulus using forced choice procedures or free-choice tests. Discrimination of emotions in presented voice samples or recognition of perceived emotion depends on the test type^(6)^.

Emotions can be described using the dimensions of valence (the continuum of pleasant versus unpleasant emotions) and arousal (the level of alertness or excitation evoked by the emotion). Anger and happiness are related to a high arousal dimension while exhibiting opposite patterns on the valence dimension: anger is recognized as negative, whereas happiness is identified as having a positive valence.

Despite substantial research regarding the acoustic properties and subjective perception of affective prosody, these variables appear to have no clear association patterns^(8)^. Most studies have focused on the arousal dimension, predominantly concerning voice intensity and pitch, with relatively few exploring valence. Overall, findings from different studies indicate that high arousal has been significantly associated with high mean F_0_ and large F_0_ variability, as well as an increased sound pressure level in the voice^(9)^. Juslin and Laukka found that strong emotion intensity in actors’ voice portrayals improved decoding accuracy. Moreover, they suggested that certain acoustic cues, such as F_0_ and high-frequency energy were highly predictive of listeners’ ratings of emotion intensity^(10)^.

The most common emotional voice samples are recordings of actors simulating vocal expressions based on emotion labels or typical scenarios^(6)^. Despite the likelihood that simulated vocal affect may result in more stereotypical and intense emotions than natural expressions, research indicates that naïve listeners do not reliably differentiate between authentic and acted-out emotional vocalizations^(11)^. Furthermore, the recognition accuracy scores for affective prosody do not significantly differ based on the speaker's status, whether they are professional actors or non-trained speakers^(11)^. Naïve listeners have demonstrated credible and consistent recognition and discrimination abilities, with an average accuracy ranging from 56% to 65% across different emotions^(6)^.

Typically, anger is comparatively better identified than joy in the voice^(6)^. Furthermore, anger and fear are recognized faster than happy vocal expressions^(5)^. According to Johnstone and Scherer^(4)^, the overall preference for negative versus positive expressions could be explained by the evolutionary advantages of signaling and perceiving vocal distress across long distances since timely detection of possible danger is critical for an organism’s survival.

### Affective prosody and individual differences

The human’s ability to understand and express emotions is essential for emotional competence and correlates with emotional intelligence^(12)^. However, like many abilities, the skills mentioned above are subject to individual differences, including age, gender, various psychosocial correlates, as well as neurobiological processing^(13)^.

A recent review by Morningstar and colleagues regarding the development of vocal affect recognition summarizes our current understanding from behavioral and neurobiological perspectives. According to behavioral studies, emotional recognition skills develop earlier in the visual domain compared to the auditory domain^(14)^. Nevertheless, vocal affect decoding skills improve throughout childhood and adolescence, eventually reaching their peak in early adulthood^(15)^.

Recent cross-sectional studies indicate that the age of the listener and the speaker may influence the capacity to decode vocal affect^(15)^. Additionally, emotional perception can follow distinct developmental trajectories. For instance, detecting pleasure and sadness may occur at earlier stages, whereas the capacity to recognize fear and surprise develops at a later stage^(15)^. However, as individuals age, a significant overall decline in their abilities to recognize vocal emotions may be observed, with potentially more pronounced deficits in identifying negative emotions^(16-18)^. In addition, these age-related deficits can be predicted by various acoustic properties and are associated with specific neural correlates^(18)^.

Gender-based differences in the recognition of emotional prosody tend to emerge during adolescence. For instance, females aged 13-15 tend to exhibit greater sensitivity to happy and sad prosody, while displaying equal sensitivity to angry vocalizations as their male counterparts^(19)^. On average, females exhibit slightly higher levels of accuracy in recognizing non-verbal emotions across different cultures, in addition to rating emotion expressions more intense and more variable than their male counterparts^(20)^. However, this advantage has raised some controversies due to inconsistent findings and alternative explanations^(21)^. As Kret and De Gelder point out, most gender-based differences have been reported in research paradigms that employ static images, while studies involving dynamic emotional stimuli have been less unequivocal^(22)^. Although research on gender-related differences in the perception of affective prosody has been relatively limited compared to visual perception, the results have been equally divergent and indicate that vocal affect recognition may be affected by both the listener’s and the speaker’s gender^(23)^.

A recent meta-analysis reported a slight overall female advantage during emotion recognition tasks moderated by a subset of factors, including age, sensory modality (visual, audio, audio-visual), valence, and the actor's gender^(24)^. Some studies that have compared emotion recognition accuracy across multiple sensory modalities indicate that female participants demonstrate higher levels of vocal affect recognition accuracy than male participants^(13,17)^.

### Affective prosody and length of the vocal stimulus

In emotion recognition studies, various vocal stimuli, ranging from short bursts to complex sentences, are used as carriers for emotional expressions. The reliability of ratings may be affected by the utterance length^(25)^. Stimulus types and their duration progressively activate emotion-specific knowledge, leading to higher accuracy and confidence ratings^(25,26)^. Listening to longer portions of an utterance tends to facilitate the process of explicit recognition and the ability to categorize the meaning of emotional prosody^(5,26)^. However, the average time needed to accurately decode emotions in speech based entirely on acoustic cues depends on the type of emotion. Vocal expressions of negative emotions require less auditory input to decode accurately, whereas expressions of happiness take much longer^(5,26)^. For example, prosodic cues conveying anger were detected from utterances lasting approximately 710 milliseconds (ms), while recognition of happiness improved at relatively long utterance durations (5-7 syllables or 977 ms)^(5)^.

Sentences are more complex syntactic units than individual words, and they include a wider range of acoustic cues. Differences in the perceived duration of the utterance, speech rate, intensity, pitch register, and prosody contour form an acoustic image of perceived emotion in listeners^(26)^. From the speaker's perspective, samples consisting of one word are too brief to encompass all the relevant cues for emotional communication, while longer samples, such as paragraphs, can be challenging for speakers to maintain emotional expression consistently throughout the speech sample^(25)^. Thus, these conditions could affect the recognition accuracy of affective emotions.

The perception of emotions can be culturally or linguistically dependent^(6)^. Previous research on the perception of emotions from vocal cues has mainly focused on German or other major languages such as English, Italian, Spanish, and others^(27)^. Encoding and decoding studies of vocal expressions of emotions have never been carried out in the Latvian language. Therefore, this study will contribute to a better understanding of decoding emotional vocal stimuli in users of a relatively small language.

The current study addressed two questions^(1)^: whether the age and gender of listeners and the length of vocal stimuli impact the discrimination accuracy of affective emotions in the voice^(2)^; whether the determined level of expression of perceived affective emotions is age and gender-dependent.

## METHODS

### Instruments and procedures

#### Voice sample recordings

Ten professional actors (5 males and 5 females) recorded nine voice samples (4 words, 4 phrases, one text sample). The male actors had a mean age of 26.6 years (SD = 4.9), and the females had a mean age of 24.6 years (SD = 1.5). None of the actors had voice disorders. All voice samples were semantically neutral. Actors recorded four words: “up”, “down”, “left”, “right” (all words have 2-syllables in Latvian); four phrases: “now press up”, “now press down”, “now press left”, “now press right” (all phrases consist of three 2-syllable words in Latvian). The paragraph included four sentences from Aesop’s Fable “The North Wind and the Sun” (Latvian version). The total length of the paragraph was 64 syllables.

Each linguistic unit was pronounced in neutral, happy, and angry intonation. Angry and happy emotions were chosen due to their distinct, high-arousal nature, representing opposite ends of the valence spectrum. Before recording, actors were prepared to express the specific emotions by reading descriptions of emotional states. For a neutral emotion: Imagine that you are currently not experiencing strong emotions and do not have specific interests or needs. Your task is to repeat the following words/phrases/text in a clear, neutral, and non-emotional voice. For a happy emotion: Imagine that you are currently very happy and content: you have been fortunate, and everything you desire has been achieved, your wishes and goals have been fulfilled, there is complete harmony between your desires, goals, and reality, all circumstances are favorable, and you are satisfied with everything. Your task is to repeat the following words/phrases/text in a clear and joyful voice. For an angry emotion: Imagine that you are currently experiencing a state of intense anger, where everything is going wrong and in contradiction to your plans, goals, and needs. You are feeling profound and powerful dislike, dissatisfaction, outrage, resentment, and frustration. Your task is to repeat the following words/phrases/text in a clear and angry voice. Each instruction was given before the recording of the specific emotion. Each voice sample was recorded three times. A pool of 810 voice samples was acquired (9 linguistic units x 3 times x 3 emotions x 10 actors).

The recording of the voice samples was carried out in a soundproof room. We used a calibrated head-worn condenser microphone AKG C520 with balanced XLR audio output (AKG Acoustics, Vienna, Austria). The microphone was placed laterally with a mouth-to-microphone angle of ± 45^0^ and a distance of ± 5 cm. The audio recordings were performed using an audio interface Scarlett Solo (Focusrite Plc. High Wycombe, United Kingdom), connected to MacBook Air. The voice signals were recorded, digitized at a sampling rate of 44.1 kHz and a resolution of 16 bits, and saved in .wav format using software PRAAT, version 6.1.31 (Paul Boersma and David Weenink, Institute for Phonetic Sciences, University of Amsterdam, The Netherlands).

The mean duration, speech rate, and fundamental frequency of recorded words, phrases, and text samples produced by the female and male actors are shown in Table 1.

**Table 1:** The mean duration, speech rate, and fundamental frequency (F_0_) of the vocal stimuli

Measure	Emotion	Words	Phrases	Paragraph
		(2 syllables)	(6 syllables)	(64 syllables)
Duration (ms)	Neutral	565	1418	16 483
	Happy	548	1275	15 831
	Angry	779	1866	16 532
Speech Rate (syllables/s)	Neutral	3.63	4.42	3.95
	Happy	3.78	4.85	4.11
	Angry	2.78	3.43	4.03
F_0_ (Hz) (females)	Neutral	201.61	202.54	202.57
	Happy	238.67	241.84	242.65
	Angry	217.94	213.69	217.91
F_0_ (Hz) (males)	Neutral	99.00	103.46	118.42
	Happy	154.90	161.02	158.58
	Angry	155.20	167.15	156.29

Caption: F_0_ = Fundamental frequency; ms = milliseconds; s = seconds

#### The selection of the voice samples

To ensure the accurate expression of the target emotion and to avoid technical recording related issues, each actor recorded every linguistic unit three times and employed various strategies. A panel of six raters was formed to choose the best voice sample from the three recorded versions by each speaker. The raters were members of the research team (all females, mean age of 44.3 years, SD 13.7 years, range 21-57 years). All three versions of each linguistic unit were played in the recorded sequence and assessed according to two criteria^(1)^: maximum reflection of the emotion (anger or happiness) or maximum non-emotional voice (in neutral voice cases)^(2)^; diction and articulation quality. Raters used a three-point scale, where 3 points were allocated to the voice sample where the selected emotions were most accurately and intensely represented. If all three entries subjectively appeared identical in the context of the emotional component, the maximum score was awarded based on pronunciation quality. If there were no noticeable differences in emotional or articulation quality context, one version had to be subjectively awarded the maximum score. In this case, the most appropriate voice sample produced by a single actor was chosen based on frequency analysis. The voice sample that received three points in most ratings was included in the set of 270 highest-ranked voice samples (90 neutral voice samples, 90 happy voice samples, and 90 angry voice samples). The selected voice samples were used as the auditory stimuli for the listening experiment.

#### The listening experiment

The experiment was designed with the open-source Python Kivy framework and presented as a mobile application on a PC tablet Lenovo TB-X606X (Lenovo Group Ltd, Beijing, China). The developed application had a user interface with two tasks on the tablet screen. The first task required the subject to choose one of three emotions after listening to the voice stimulus. The second task was to evaluate the intensity of the emotion in a voice sample using a 100 mm horizontal visual analog scale (VAS). The scale has anchors at both ends labeled “minimal intensity of emotion” and “maximal intensity of emotion”.

To minimize the potential perceptual aftereffects or interference resulting from the mixing of different types of emotional valence stimuli, all 270 selected standard voice samples were presented in a blocked sequence and mixed with distractors or oddball stimuli within each block. The oddball stimuli (five angry, five happy, five neutral) were voice samples that received lower scores from the raters’ panel. By implementing this experimental design, we aimed to increase the evaluation accuracy of the emotional intensity within each type of emotion.

The experiment consisted of three randomly shuffled experimental blocks (i.e., one participant listened in angry-happy-neutral order, another happy-angry-neutral, a third neutral-angry-happy, etc.). Each block contained 100 stimuli (for example, 90 standard stimuli (happy voice) and ten distractors (neutral, angry voice)) presented in a pseudo-random order. Distractors were distributed with a probability of 10%, i.e., one distractor within ten standard stimuli distributed pseudo-randomly. Thus, in one instance, the distractor pops up as third out of ten, in another, as seventh out of ten, and so on. However, no distractors were presented immediately after one another.

Participants were explicitly informed about the distractors and were instructed to pay attention and evaluate each stimulus as a potential deviant. In addition, this experimental design increased the likelihood that participants were constantly attentive and selective throughout the experiment, which lasted for at least one hour.

Before beginning to listen, the participants were introduced to the same emotional state descriptions presented to the actors before the voice samples’ recordings. A short practice session was provided for all of them. There was no time limit on the task, and participants were allowed to take a short break whenever necessary. Participants used AKG K240 headphones (AKG Acoustics, Vienna, Austria) and a touchscreen pen to complete the experimental tasks. All responses were automatically saved as a text file (.csv).

### Participants

Respondents were included in the study based on the criterion of Latvian being their native language. Thirty-two female and male age-matched participants (16 males and 16 females) were invited to participate in the study on a volunteer basis. The mean age for males was 29.3 years (SD = 12.1 years; range = 18-59 years). The mean age for females was 28.8 years (SD = 12.3 years; range = 19-58 years). The participants represented a cross-section of educational backgrounds, with nine having completed secondary education, fourteen possessing higher education qualifications, and nine being current students; notably, none held expertise in speech sciences. Prior to the experiment, a hearing test was performed at frequencies from 0.25 to 8 kHz (mod. AD 226; Interacoustics, Middelfart, Denmark), as hearing impairments were a basis for exclusion from the study. No participants had hearing disorders. All respondents involved in the study had signed the Informed Consent Form.

### Statistical analysis

Statistical analysis was done using SPSS software (v. 28; SPSS Inc., New York, NY). The frequency analysis was used to select one of the three-voice samples with the highest intensity of expressed emotion. The Shapiro-Wilk test for small sample sizes was applied to determine the distribution of all obtained data. The analysis showed that data were not normally distributed, so nonparametric statistical methods were used for further data analysis. The Related-Samples Friedman’s Two-way analysis method with the Post Hoc Kruskal-Wallis test where Bonferroni corrections were applied was used to investigate differences in the number of correctly identified emotional valences. The Related Samples Friedman’s two-way analysis test and Post Hoc analysis with Wilcoxon Signed rank test with the Bonferroni correction applied demonstrated a statistically significant difference in discrimination accuracy depending on the type of linguistic unit. The Mann-Whitney test was used to investigate gender differences in discriminating accuracy of emotional valence and perception of the intensity of the specific emotion in the voice. Spearman's correlation methods were used to determine associations between investigating variables (discriminating accuracy and perception of emotion intensity) and age.

The Ethical Committee for Clinical Research of the P. Stradins Clinical University Hospital (Riga, Latvia) approved the study (No. 220222-11L).

## RESULTS

### Discrimination accuracy of emotions in voice

Thirty-two age-matched naïve participants listened to words, phrases, and short texts recorded by ten professional actors whose task was to imitate neutral, happy, and angry emotions in the given linguistic units. Each participant listened to 300 voice samples. However, only voice samples selected by the experts (N = 270) were analyzed, and the distractors (N = 30) were excluded from the analysis. In total, 8640 responses were further processed. Participants recognized neutrally expressed voice samples at a rate of 73.2%, while happy and angry voices were identified by 67.4% and 84.9% of listeners, respectively (Table 2). An equal number of voice samples represented each emotion. The Related-Samples Friedman’s Two-way analysis method with Post Hoc Kruskal-Wallis test where Bonferroni corrections were applied was used to investigate whether there were differences in the number of correctly identified emotions. We found a statistically significant difference between the number of correctly identified angry and happy voices (*Z* = -0.913, *p* < .001). However, no significant differences were found between the correctly identified neutral and angry emotions and neutral and happy emotions. (*Z* = -0.500, *p* = .137; *Z* = 0.453, *p* = .210).

**Table 2:** Response matrix of emotional valence in voice samples (in %)

Voice stimuli	Neutral	Happy	Angry
Neutral	73.2	7.9	18.9
Happy	25.6	67.4	7.0
Angry	13.8	1.3	84.9

The study found that affective emotions were often misidentified as neutral and vice versa (happy – neutral, angry – neutral). Happy emotions were perceived as neutral rather than neutral being perceived as happy (*Z* = -3.735, *p* < .001). We found no statistically significant difference between perceiving neutral emotions as angry and vice versa (*Z* = -1.304, *p* = .192).

The Mann-Whitney test revealed no gender differences in the correctly discriminated emotions in the voice samples. Thus, for neutral emotions *U* = 128.5, *p* = .985, for happy emotions *U* = 127.0, *p* = .970, and for angry emotions *U* = 110.5, *p* = .508.

**Figure 1:**
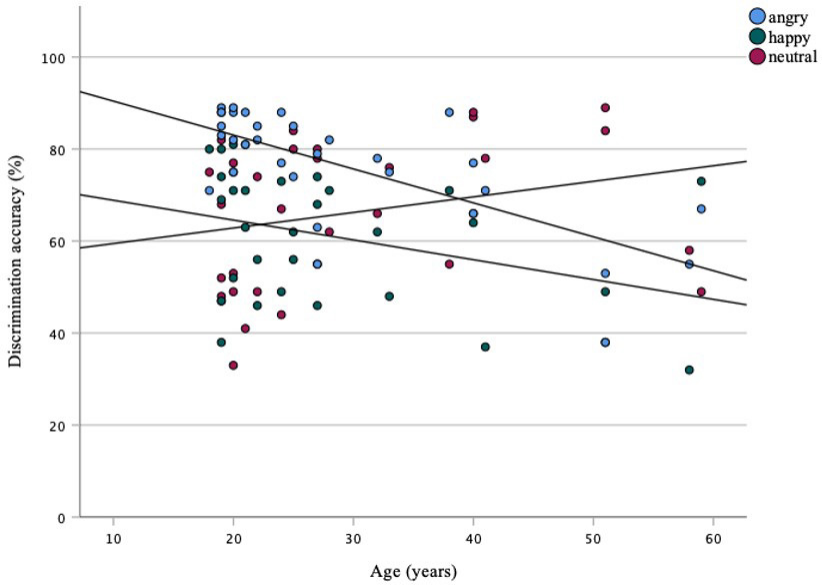
Relationship between discrimination accuracy of angry, happy, and neutral emotions and age

Furthermore, the Spearman rank-order correlation was run to determine the relationship between discrimination accuracy and listeners’ age. There was a strong, negative correlation between the discrimination accuracy of angry voice stimuli and listeners' age (*r_S_* = -.642, *p* < .001). A weak negative correlation was found between the discrimination accuracy of happy stimuli and age (*r_S_* = -.364, *p* = .041), and a weak, positive correlation was found between the age of listeners and discrimination accuracy of neutral stimuli (*r_S_* = .374, *p* = .035) (Figure 1).

Caption: Lines denote regression lines for separate vocal emotions. Symbols indicate correctly discriminated emotions of single participants

**Figure 2:**
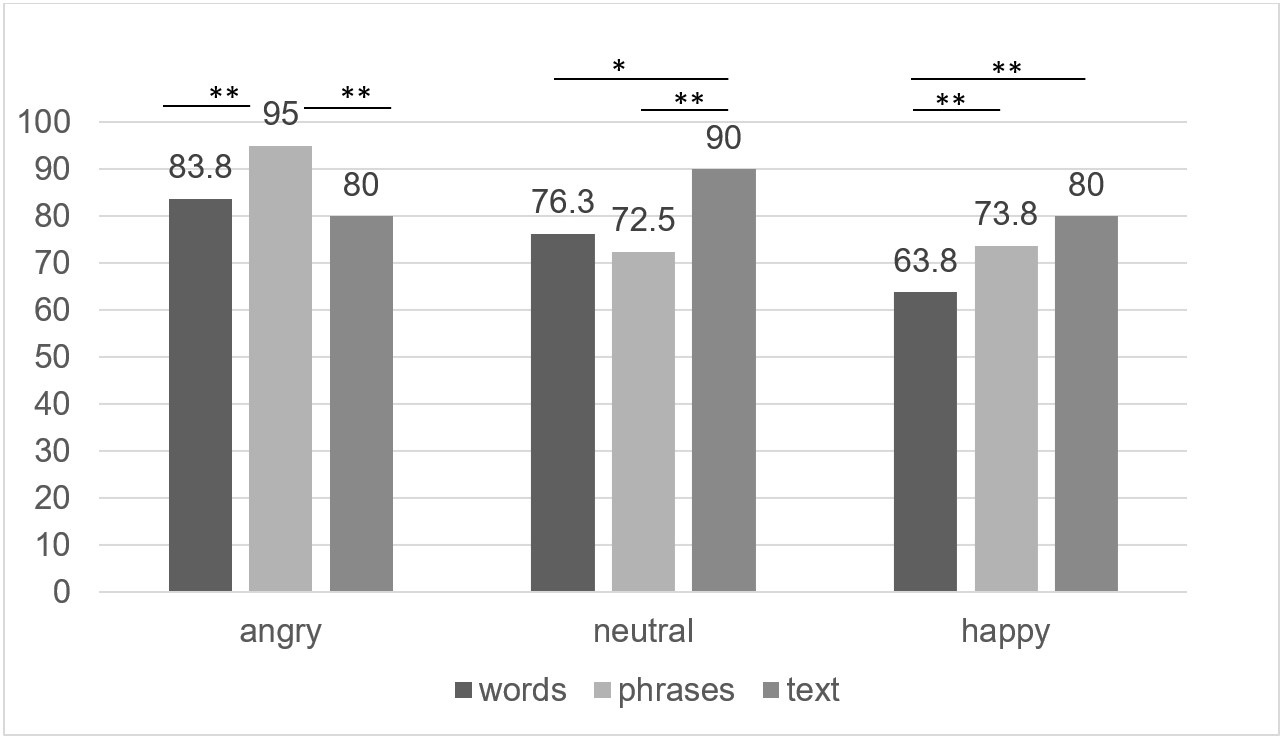
Median values of discrimination accuracy of angry, neutral, and happy emotions in words, phrases, and text

The Friedman test was conducted to analyze discrimination accuracy based on type of stimuli. Pairwise comparisons were made with a Bonferroni correction for multiple comparisons. For all types of emotions, discrimination accuracy varied significantly across different linguistic units: for angry voices *χ^2^*^(2)^ = 24.402, *p* < .001; for neutral voices *χ^2^*^(2)^ = 14.992, *p* < .001, and for happy voices *χ^2^*^(2)^ = 36.049, *p* < .001. Post hoc analysis revealed statistically significant differences in discrimination accuracy of angry voices between phrases (*Mdn* = 95.0, *IQR* = 16.2) and words (*Mdn* = 83.8, *IQR* = 19.4) (*p* < .001) and phrases and paragraphs (*Mdn* = 80.0, *IQR* = 30.0) (*p* < .001), but not between words and paragraphs (Figure 2). There were statistically significant differences in discrimination accuracy of neutral voices between paragraphs (*Mdn* = 90.0, *IQR* = 40.0) and words (*Mdn* = 76.3, *IQR* = 31.9) (*p* = .037) and paragraphs and phrases (*Mdn* = 72.5, *IQR* = 31.9) (*p* = .001), but not between words and phrases. For happy voices, statistically significant differences in discrimination accuracy were found between words (*Mdn* = 63.8, *IQR* = 22.5) and paragraphs (*Mdn* = 80.0, *IQR* = 17.5) (*p* < .001) and between words and phrases (*Mdn* = 73.6, *IQR* = 26.9) (*p* < .001), but not between paragraphs and phrases.

Caption: *p < .05; **p < .01

There were weak correlations between age and discrimination accuracy of neutral emotions in words (*r_S_* = .367, *p* = .039) and happy emotions in phrases (*r_S_* = -.358, *p* = .044). Strong negative associations were found between age and angry emotions expressed in words, phrases, and paragraphs (*r_S_* = -.592, *p* < .001; *r_S_* = -.612, *p* < .001; *r_S_* = -.642, *p* < .001).

### Determining the intensity level of affective emotions expressed through the voice

**Figure 3:**
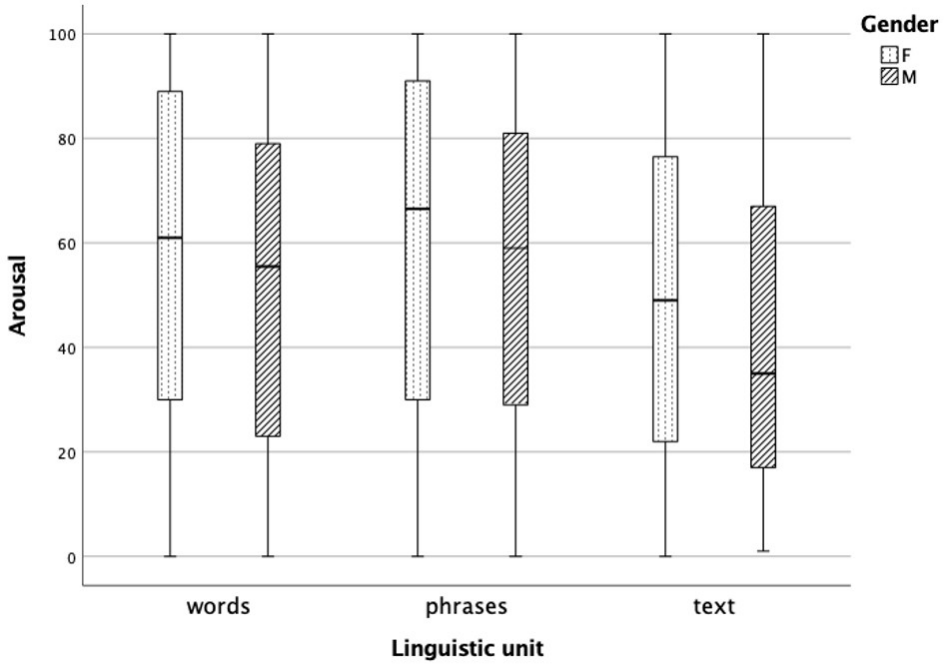
The perception of angry emotion intensity in words, phrases, and text in females (n = 16) and males (n = 16)

The intensity of perceived emotions was rated for samples of angry and happy voices. Further analysis only included emotions that correctly matched the target emotion. A Mann-Whitney U test was conducted to compare intensity ratings between males and females. Each linguistic unit was analyzed separately. Females rated perceived angry emotions higher than males across all linguistic units, that is words (*z* = -3.599, *p* < .001), phrases (*z* = -3.218, *p* = .001), and text samples (*z* = -2.272, *p* = .023) (Figure 3).

**Figure 4:**
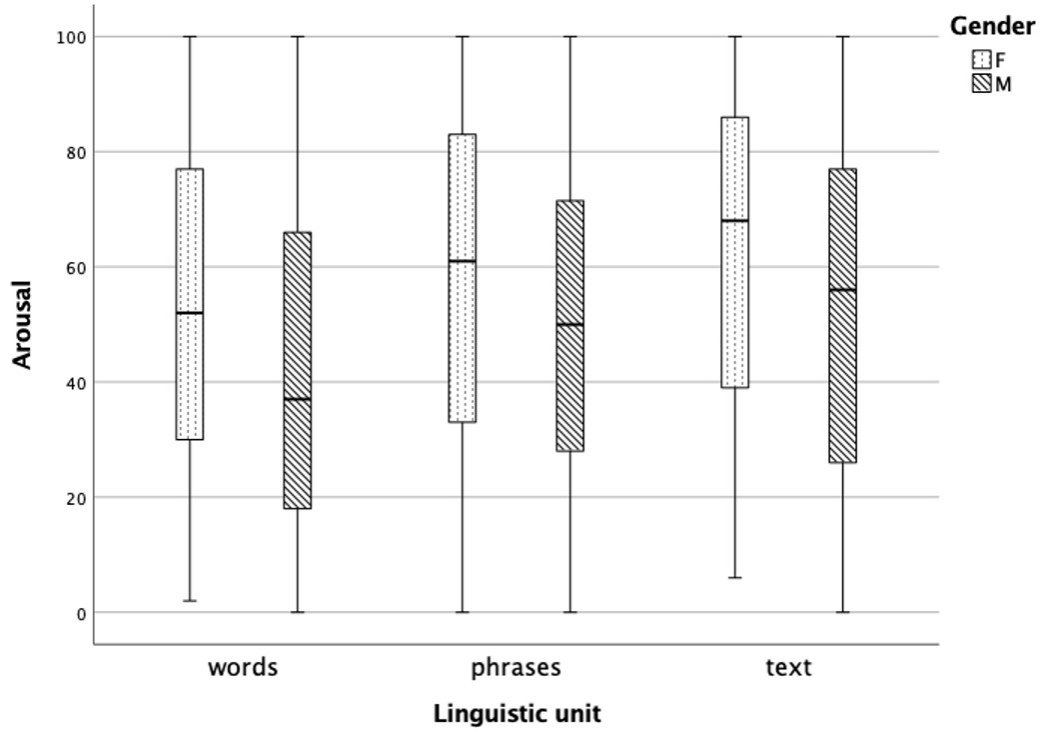
The perception of happy emotion intensity in words, phrases, and text in females (n = 16) and males (n = 16)

Figure 4 demonstrates that females perceive happy emotions more intensely than males in words, phrases, and text. Median scores for happy emotions intensity were statistically significantly higher in females than in males for all the linguistic unit types, that is words (*z* = -5.799, *p* < .001), phrases (*z* = -4.706, *p* < .001), and text samples (*z* = -2.699, *p* = .007).

A Spearman's rank-order correlation found no association between the perceived intensity of emotions and participants’ age for both happy and angry emotions in both males and females. Females: happy emotions *r_S_* = -.391, *p* = .134, angry emotions *r_S_* = -.402, *p* = .123. Males: happy emotions *r_S_* = -.019, *p* = .944, angry emotions *r_S_* = -.040, *p* = .884.

## DISCUSSION

This study aimed to determine how the age and gender of listeners and the type of vocal stimuli affect the accuracy of emotion discrimination in voice. Accuracy was measured as the percentage of accurate attributions (the decoded emotion) given a categorical criterion (the encoded emotion)^(3)^. Physiological arousal, triggered by an emotional stimulus.

This was an emotion-decoding study that examined the ability of participants to identify emotions based on their perception of the expressed auditory features and the inferences they drew from them^(3)^. Using a forced-choice procedure, the participants were asked to select one of three voice options - angry, happy, or neutral. According to Scherer, this type of emotion selection should be separated from an emotion recognition task, requiring the participant to recognize a particular category in its own right without any cues^(6)^. Previous studies have shown that the recognition of emotions from standardized voice samples, using renderings from actors, attains 74% accuracy for neutral emotions, 77% for angry emotions, and 57% for happy emotions^(6)^. The results of our study showed that the accuracy score for discriminating a neutral voice (73%) remained the same as that in previous studies despite using a forced-choice procedure^(6)^. However, the accuracy of discriminating affective emotions in our study was higher, with almost 85% accuracy for angry and 67% accuracy for happy emotions. Participants may have identified affective emotions more accurately due to the limited options provided, while in emotion recognition tasks, they were not required to make choices between alternatives. According to the current study, anger was found to have the highest discrimination accuracy score. This is in line with several studies^(3,16,23)^ that have found that anger can be more accurately recognized through its unique acoustic profile, combined with a biological ability to sense potential danger or high arousal. Like other studies, we found a neutral tone easier to discriminate than happy emotions in speech^(6,16,23)^.

The results demonstrated that the discrimination accuracy of emotions in the voice samples was more listeners’ age than gender dependent. The absence of observed gender differences in the study could be attributed to the modality of stimuli used. Previous research has identified a more pronounced female advantage in tasks with combined audio and visual cues than in audio-only stimuli^(24)^. As our study relied on voice stimuli, this constrained the potential for detecting gender-specific patterns in emotion discrimination accuracy. Additionally, a possible ceiling effect in emotion-detecting tasks may have contributed to the non-significant gender findings. High performance across participants can obscure subtle differences, suggesting that the experimental task was potentially too simple (only discrimination between angry, neutral, and happy emotions) to capture the finer gradations of emotional discrimination abilities between genders. Valence and arousal are somewhat interdependent dimensions (e.g., highly negative stimuli are usually perceived as excitatory). Both fear and anger are commonly classified as high in arousal and unpleasant; therefore, these emotional categories can be more challenging to distinguish and separate. The more opposed an emotion is in both dimensions (valence and arousal), the easier it is to discriminate them.

The study results show that young adults are better at identifying affective, emotional tones than middle-aged adults. These results align with previous studies stating that the ability to recognize emotions decreases steadily throughout the lifetime^(17)^. In fact, emotional speech recognition begins to decline in middle-aged participants^(16)^. In our study which included young and middle-aged adults, similarly to Paulmann et al.^(16)^, we found that younger participants generally had higher emotion recognition rates. Older adults differentiated positive (happiness) and negative (anger) emotions less precisely with a trend to neutralize them^(17)^.

The difference in emotion discrimination accuracy between young and middle-aged adults may be attributed to the varied use of acoustic cues^(16)^, different experiences in social interactions, or a decrease in sensory acuity in older participants during a listening task^(17)^. Fundamental frequency contour, amplitude, timbre, and temporal aspects describe emotions' acoustic profiles and support emotion processing and recognition^(10)^. Studies indicate that young and middle-aged adults may be processing the acoustic cues of stimuli differently, and middle-aged listeners experience trouble using acoustic input to categorize specific emotions^(16)^. Additionally, age differences in emotion discrimination accuracy may be explained by communication experience. Middle-aged adults with more communication experience may have encountered more variations of neutral speech that border on angry or happy but may still be considered neutral. For instance, a voice stimulus perceived as angry by a young listener was deemed neutral but with a threatening tone by an older participant. However, differences in discrimination accuracy between respondents of different ages may also be due to the organization of the experiment’s procedure. Participants listened and rated 300 voice stimuli, requiring approximately one hour of concentration. Sustained attention and working memory are critical for emotion recognition tasks^(17)^. These abilities decrease with age and impact the performance of the given tasks.

In addition to the reasons discussed above, the authors want to add that the age of the actors/speakers (mean age of 26.6 and 24.6 years for males and females) may also have contributed to the difference in discrimination accuracy of emotions between young and middle-aged adults. A similar observation was found in the Sen et al.^(28)^ study, where young listeners better recognized angry, happy, and neutral emotions expressed by young speakers. Expression style, familiarity with that style, and motivation to engage with the same-age speakers facilitated emotion recognition accuracy.

Our research has shown that the type of voice stimulus impacts the accuracy of discriminating vocal expressions of emotions. Angry emotions were more accurately recognized in phrases, but happy and neutral voice tones had better discrimination accuracy in paragraph samples. The results aligned with previous studies suggesting that angry emotions were identified through significantly less acoustic information than happiness^(5)^. The discrimination accuracy of happy emotions differed significantly between words, phrases, and texts, and the highest average discrimination accuracy score was observed in texts. Longer speech segments that include more syllables improve the recognition of vocal emotions^(5,26)^. Five to seven-syllable utterances enhance happiness recognition^(5)^.

Speech that conveys emotions typically shows differences in voice quality, pitch patterns, and timing at the phrase level. Additionally, the distinctive acoustic features associated with recognizing basic emotions in speech are most accurately decoded and consciously identified when processing three to four spoken syllables^(26)^. Moreover, longer stimuli are considered more ecologically valid because they encompass a broader range of acoustic cues. Therefore, it was unsurprising that neutral and affective emotions were more easily recognized in phrases and paragraphs than in individual words.

Accuracy in discriminating angry emotions was higher for phrases compared to paragraphs. This observation supports the notion that decoding negative emotions in vocal expressions requires less auditory input^(26)^. This could be attributed to the evolutionary inclination to respond more promptly to short phrases rather than to extended connected speech during emergencies. Additionally, in longer speech segments, the acoustic characteristics associated with angry emotions may become diffused, making it challenging to distinguish between the three defined emotion types, as extended stimuli might contain increased variations. Schuller et al.^(29)^ emphasized that in real-life conversations, it is essential to estimate the emotion before a longer utterance is finished.

The second aim of this study was to investigate the impact of age and gender on how happy and angry emotions are perceived by listeners. The results revealed that age did not affect the perceived intensity of emotions. Significant differences in judgments about perceived emotion intensity were observed between male and female participants. Despite all participants hearing the same vocal stimuli, females rated anger and happiness intensity higher than males. The trend was observed in all types of presented vocal stimuli, including words, phrases, and text.

Studies conducted so far have focused more on gender differences in decoding vocal emotions, showing that females are more accurate than males^(23)^. However, these studies reveal a gap regarding the perception of emotional vocal expression intensity. The level of emotional expression is crucial in human communication because intensifying or weakening an emotion makes the message more nuanced and precise. In order to perceive these nuances, the listener must be endowed with emotion perception competence, the ability to accurately perceive and interpret the emotional state of other individuals so as to correctly infer their reactions to salient events and to predict their action tendencies^(13)^.

Our research shows that females rate emotions of happiness and anger significantly higher than males. Various theories attempt to explain gender differences in emotion perception. From a phylogenesis perspective, women tend to have social-emotional roles like childcare, romantic relationships, and organizational responsibilities, which make them more skilled at identifying subtle emotional cues^(21)^.

The hypothesis of emotional sensitivity suggests that women are more capable of recognizing and accurately identifying emotions due to their emotional reactivity speed, a negative bias for experiencing affect, or general emotional hypervigilance^(30)^. In contrast, men are less competent in emotion perception than women and are more uncertain about their perception. They may easily get confused when rating the intensity of different emotions and think of themselves as less confident in perceiving, understanding, and regulating emotions^(21)^.

Finally, this study focused on the discrimination accuracy of vocal expressions of emotions or decoding quality of perceived voice signals. Although the study's results highlighted the percentage of correctly decoded emotions, our attention was drawn to cases of misidentified emotions. Miscommunication occurs when the transmission of information breaks down^(1)^. The study’s results lead us to consider Titze’s claim that elements of the voice signal can impact the decoding process and increase entropy in daily communication^(1)^. Moreover, Scherer et al.^(3)^ highlighted that the specification and investigation of additional acoustic parameters concerning emotions would be essential to our future understanding of vocal expression and perception of emotions. The investigation of the acoustic properties of the voice signals underlying the expression of emotions and which impact the decoding process will be the task of future research.

Although the study has employed professional actors to ensure consistency and quality in the emotional expressions presented, we recognize this as a potential limitation. The intensity and stereotypical nature of portrayed affective emotions can inherently differ from those experienced in everyday life. These methodological choices may affect the ecological validity of the findings related to how listeners perceived happy and angry emotions. Additionally, the authors acknowledge the omission of data about listeners' socioeconomic status, which limits understanding of its potential influence on emotion discrimination accuracy and perception of the intensity of affective emotions.

## CONCLUSION

The perception of the vocal expression of emotions varies by age and gender. The listeners’ age affects the discrimination accuracy of happy, angry, and neutral emotions, while the perception of the intensity level of affective emotions is more gender related. Young adults are better at distinguishing happy and angry emotions than middle-aged adults. Middle-aged adults tend to categorize perceived affective emotions as neutral. Females rate the expression of affective emotions in voices higher than males. In addition, the length of the voice stimuli impacts the accuracy of emotion discrimination.
